# Patients with obstructive sleep apnea have suppressed levels of soluble cytokine receptors involved in neurodegenerative disease, but normal levels with airways therapy

**DOI:** 10.1007/s11325-020-02205-y

**Published:** 2020-10-09

**Authors:** Ye Wang, Richard B. Meagher, Suresh Ambati, Ping Ma, Bradley G. Phillips

**Affiliations:** 1 Department of Statistics, University of Georgia, Athens, GA 30602 USA; 2 Department of Genetics, University of Georgia, Athens, GA 30602 USA; 3 Clinical and Administrative Pharmacy, University of Georgia, Athens, GA 30602 USA; 4 Clinical and Translational Research Unit, University of Georgia, Athens, GA 30602 USA

**Keywords:** Obstructive sleep apnea, OSA, Cytokines, Airways therapy, CPAP, Neurodegenerative disease, Microglia

## Abstract

**Purpose:**

Obstructive sleep apnea (OSA) results in systemic intermittent hypoxia. By one model, hypoxic stress signaling in OSA patients alters the levels of inflammatory soluble cytokines TNF and IL6, damages the blood brain barrier, and activates microglial targeting of neuronal cell death to increase the risk of neurodegenerative disorders and other diseases. However, it is not yet clear if OSA significantly alters the levels of the soluble isoforms of TNF receptors TNFR1 and TNFR2 and IL6 receptor (IL6R) and co-receptor gp130, which have the potential to modulate TNF and IL6 signaling.

**Methods:**

Picogram per milliliter levels of the soluble isoforms of these four cytokine receptors were estimated in OSA patients, in OSA patients receiving airways therapy, and in healthy control subjects. Triplicate samples were examined using Bio-Plex fluorescent bead microfluidic technology. The statistical significance of cytokine data was estimated using the nonparametric Wilcoxon rank-sum test. The clustering of these high-dimensional data was visualized using *t*-distributed stochastic neighbor embedding (t-SNE).

**Results:**

OSA patients had significant twofold to sevenfold reductions in the soluble serum isoforms of all four cytokine receptors, gp130, IL6R, TNFR1, and TNFR2, as compared with control individuals (*p* = 1.8 × 10^−13^ to 4 × 10^−8^). Relative to untreated OSA patients, airways therapy of OSA patients had significantly higher levels of gp130 (*p* = 2.8 × 10^−13^), IL6R (*p* = 1.1 × 10^−9^), TNFR1 (*p* = 2.5 × 10^−10^), and TNFR2 (*p* = 5.7 × 10^−9^), levels indistinguishable from controls (*p* = 0.29 to 0.95). The data for most airway-treated patients clustered with healthy controls, but the data for a few airway-treated patients clustered with apneic patients.

**Conclusions:**

Patients with OSA have aberrantly low levels of four soluble cytokine receptors associated with neurodegenerative disease, gp130, IL6R, TNFR1, and TNFR2. Most OSA patients receiving airways therapy have receptor levels indistinguishable from healthy controls, suggesting a chronic intermittent hypoxia may be one of the factors contributing to low receptor levels in untreated OSA patients.

**Electronic supplementary material:**

The online version of this article (10.1007/s11325-020-02205-y) contains supplementary material, which is available to authorized users.

## Introduction

Obstructive sleep apnea (OSA) is a sleep-related breathing disorder associated with numerous adverse health effects. OSA patients may display any one or several symptoms including fragmented sleep, snoring, excessive daytime sleepiness, fatigue, high blood pressure, irritability, depression, memory loss, and loss of concentration. Fragmented sleep patterns, abnormally long pauses in breathing, or abnormally low levels of breathing during sleep result in poor oxygenation of the blood and chronic intermittent tissue hypoxia. Hypoxia leads to tissue inflammation, which appears to be one major cause of the diseases associated with OSA and OSA’s increased mortality risk. These health problems develop over months and years and include cardiovascular disease, metabolic syndrome, kidney disease, autoimmune diseases, and the focus of this study, neurodegenerative disease (ND). OSA has been associated with the increased risk and severity of the symptoms of Alzheimer’s disease [1, 2], amyotrophic lateral sclerosis [3], Parkinson’s disease [4, 5], multiple sclerosis [6, 7], schizophrenia [8, 9], depression disorders [10, 11], and cognitive dysfunction [12, 13]. Continuous positive airways pressure therapy (CPAP) is the mainstay of OSA treatment because it improves oxygenation, reduces inflammation, and CPAP is shown to reverse many symptoms and risks associated with OSA, including ND [5, 14–21]. Dental airway devices produce a similar treatment effect and have gained some acceptance as an effective alternative to CPAP [22, 23]. The success of CPAP treatment further supports a possible role for hypoxia-induced inflammation in ND risk. Unfortunately, a significant portion of patients find nightly CPAP therapy intolerable and reported non-compliance rates are high, ranging from 10 to 40% [24–28]. Hence, there is a pressing need for pharmaceutical treatments that might amend CPAP.

Brain inflammation is common to most NDs. A cytokine-centric model of neurodegeneration predicts that strong peripheral inflammation results in increased levels of inflammatory leukocytes and cytokines infiltrating the central nervous system (CNS), where they initiate neuroinflammation and neurodegeneration [29, 30]. Inflammatory monocytes and macrophages and cytokines in the brain activate microglial cells. Activated microglia secrete their own factors and some of the same cytokines that direct neuronal death and increase in the permeability of the blood brain barrier (BBB) to amplify the problem [30]. Elevated levels of two pro-inflammatory cytokines, in particular, tumor necrosis factor alpha (TNF) and interleukin 6 (IL6), are observed in patients with Alzheimer’s disease [31], amyotrophic lateral sclerosis [32, 33], multiple sclerosis [34], Parkinson’s disease [35–37], depression disorders [38, 39], and acute patients with schizophrenia or bipolar disorder [40, 41]. Elevated expressions of both TNF [42, 43] and IL6 [44, 45] are strongly associated with microglial activation, neuroinflammation, and neurodegeneration. Hence, their signaling appears central to many NDs.

The molecular mechanisms by which OSA increases the risk of various NDs and behaviorial symptoms such as irritability, depression, memory loss, and loss of concentration are poorly understood. Untreated OSA patients all suffer from chronic intermittent hypoxia. Hypoxia-induced oxidative stress signaling is known to alter the otherwise balanced levels of a number of pro-inflammatory and anti-inflammatory cytokines [46]. Elevated levels of TNF and IL6 are observed in cultured cells treated with mild hypoxia [47, 48], in the carotid body of mice treated with chronic hypoxia [49], in the ischemic rodent brain [50], and in most patients with OSA. In a majority of studies examining TNF levels, OSA patients have shown 1.2- to 2.5-fold higher serum levels [51] relative to control subjects. In some, but not all studies, OSA patients also show modest increases in IL6 [52–58]. CPAP treatment of OSA patients was most often, but not always, associated with normal levels of these cytokines similar to the levels in control subjects [52, 59–69]. The normal levels of TNF and IL6 observed in most OSA patients receiving airways therapy and the generally protective role of airways therapy to patient health strongly support the idea that chronic intermittent hypoxia induces inflammatory cytokines that are causal to neuroinflammation and ND.

The goal of this study was to identify other neuroinflammatory cytokines in serum whose expression levels were altered in OSA patients and but normal with CPAP treatment, focusing on TNF receptors, TNFR1 and TNFR2, and the IL6 receptor IL6R and its co-receptor gp130. To date, neither large increases nor large decreases in the expression of the soluble isoforms of these cytokine receptors have been associated with OSA. IL6 receptor complexes may be composed of soluble IL6, the membrane-bound receptor isoform or soluble isoform of IL6R, and the membrane-bound receptor or soluble isoform of co-receptor gp130 [70–77]. IL6 receptor complexes are central to inflammatory signaling, neurodegeneration, and ND [70–77]. The soluble, non-membrane-bound, isoforms of gp130 and IL6R both modulate inflammatory IL6/IL6R/gp130 membrane signaling and both are capable of attenuating IL6 signaling [78–80]. Altered serum levels of the soluble isoforms of two TNF receptors, tumor necrosis factor receptor TNFR1 (*TNFRSF1A*) and TNFR2 (*TNFRSF1B*), are linked to inflammatory signaling, neuronal cell death, and regeneration, and altered in patients with ND [29, 32, 81–97]. The soluble, non-membrane-bound, isoforms of these cytokine receptors can significantly antagonize signaling by IL6 and TNF, respectively [98–100]. Changes in the expression of these cytokine receptors have the potential to increase or decrease neuroinflammatory signaling by IL-6 and TNF, and hence, contribute to ND risk. The serum levels of the soluble isoforms of gp130, IL6R, TNFR1, and TNFR2 were significantly lower in OSA patients relative to control subjects, but OSA patients receiving airways therapy had levels indistinguishable from controls.

## Materials and methods

### Study subjects

A total of 46 study subjects were enrolled in the study following informed consent, including nineteen subjects with untreated OSA (i.e., not on airways therapy) and nineteen treated OSA individuals, who were diagnosed by polysomnography using the Apnea Hypopnea Index (AHI) (Table 1) [101, 102]. The airway-treated OSA subjects recorded using primary CPAP, except for one patient who used a dental airways device, and both devices had been employed for more than 6 months [22, 23]. Eight control individuals were recruited that had undergone polysomnography and did not have sleep disordered breathing. Subject characteristics (age, gender), anthropometrics (weight, weight, BMI), history of CVD, medication history, AHI, SaO_2_, and ESS (Epworth Sleepiness Scale) as well as fasting cholesterol, glucose, and HS-CRP were evaluated in all subjects [54, 103–105] are shown in Table 1. Treated and untreated OSA patients are well matched for nearly all parameters, while the control group was considerably younger and leaner, and presumably represented an optimal cytokine levels for comparison.

**Table 1:** Patient biometric and laboratory data

	Control subjects (*n* = 8)	Airway-treated patients (*n* = 19)	Apneic patients (*n* = 19)
Female/male	6/2	7/12	7/12
Age	37.7 ± 12.1	60.6 ± 10.5	58.2 ± 12.4
Hypertension or heart disease Y/N	2Y/6N	11Y/8N	11Y/8N
Race C/H/B(A/M)/A	4C/0H/3B/1A	17C/0H/2B/0A	11C/1H/6B/1A
BMI	25.7± 5.21	33.1 ± 9.07	35.0 ± 9.22
AHI at time of diagnosis	1.58 ± 1.64	35.7 ± 24.8	26.8 ± 25.7
SaO_2_ low %	92.4% ± 1.92%	80.7% ± 5.4%	76.4% ± 10.3%
ESS	6± 3.30	7.16 ± 5.80	7.94 ± 4.02
Glucose mg/dL	94.4± 9.21	106 ± 18.7	104 ± 12.1
Cholesterol mg/dL	170± 23.8	181 ± 25.1	179 ± 43.1
HDL mg/dL	53.8± 14.7	51.8 ± 18.1	45.7 ± 17.3
LDL mg/dL	99.2± 22.4	104 ± 27.6	106 ± 35.8
hs-CRP mg/L	1.07 ± 0.736	4.84 ± 7.65	3.48 ± 5.60
Chronic meds Y/N	2Y/6 N	16Y/3 N	16Y/3N

Race (*C* Caucasian, *H* Hispanic, *B* African American, *A* Asian); *BMI* Body Mass Index, *ESS* Epworth Sleepiness Score, *AHI* Apnea Hypopnea Index, *HDL* high-density lipoprotein, *LDL* low-density lipoprotein, *hs-CRP* high-sensitivity C-reactive protein. Standard errors are indicated where appropriate

### Powering the study

The null hypothesis being tested was that OSA patients and airway-treated OSA patients would express the same levels of soluble cytokine receptors. The alternate hypothesis being tested was that OSA patients would express higher or lower levels of soluble cytokine receptors relative to the airway-treated OSA patients [106]. These hypotheses were tested using Bio-Plex system to assay cytokine receptor levels, which provides greater sensitivity, a wider dynamic range, greater effect sizes, and more statistical significance for each assay than conventional cytokine receptor immunoassays used in most previous studies. In sample size planning, the potential for large effect sizes for the differences in the levels of the four cytokines examined and study costs were considered [107, 108]. Preliminary data showed large effect sizes for cytokine receptor levels with high levels of statistical significance (see statistical analysis). Hence, it was concluded that modest OSA and airway-treated OSA patient sample sizes would be sufficient to power a statistically significant preliminary study that would avoid both type I (false positive) and type II (false negative) errors [108] as recommended by the Federal Food and Drug Administration’s study guidelines for estimating minimum appropriate human subject sample size [109].

### Cytokine receptor assay

The levels of soluble inflammatory cytokine receptors in serum were examined using multiplex kits that quantify biomarkers of human inflammation (Bio-Rad #171AL001M). The 96-well plates were assayed using a Bio-Rad Bio-Plex instrument. The Bio-Plex system has the advantage that hundreds of beads estimate each cytokine level in each well, which improves the statistical accuracy of each individual well estimate. All the serum samples, standards, and assay controls were prepared as per the manufacturer’s instructions (Bio-Rad Bulletin 10044281). As recommended, each serum sample was diluted fourfold. Fifty microliters of this dilution was run in triplicate for the 8 control, 19 untreated OSA patients, and 19 airways therapy–treated OSA patients instead of duplicate samples recommended by the manufacturer, in order to better estimate experimental errors for each cytokine measurement. The picogram per milliliter output data for each serum cytokine receptor level was normalized to the concentration of standards, run as eight times fourfold dilutions of each cytokine, and run in duplicate on each plate. The quantitative nature of these assays over the expected concentration ranges estimated for serum samples was confirmed by comparing the fluorescence output of quadruplicate standard samples run in eight steps of a fourfold dilution series. The standard error of the lowest concentration standards used to estimate concentration was less than 15% and much less than that for higher concentrations.

### Management of data sets and statistical analysis

The data output from separate Bio-Plex plates were combined to make a single Excel data file, with one sheet for each cytokine receptor and separate sheets for biometric data (see Supplemental Data File 1). At this point, the data were moved into R v3.5.1 for further statistical analysis. The data for individual human subjects were visualized using Boxplot. After applying the Kolmogorov–Smirnov test, it was obvious that the airway-treated patient data for each the cytokine were not normally distributed (*p* value < 0.05) and generally fell into two groups of values. The Kolmogorov–Smirnov test is a nonparametric goodness-of-fit test and could be used to determine whether an underlying probability distribution differs from a hypothesized distribution. Therefore, the nonparametric Wilcoxon rank-sum test was used to estimate *p* values for the significance of pairwise differences in cytokine receptor levels among OSA patients, airway-treated OSA patients, and controls. To visualize the high-dimensional data for the levels of all four cytokine receptors among all patients and controls in a two-dimensional map, the nonparametric t-SNE visualization method [110] was applied using the Rtsne statistical package available online [111].

## Results

###  Serum cytokine receptor levels 

**Fig. 1:**
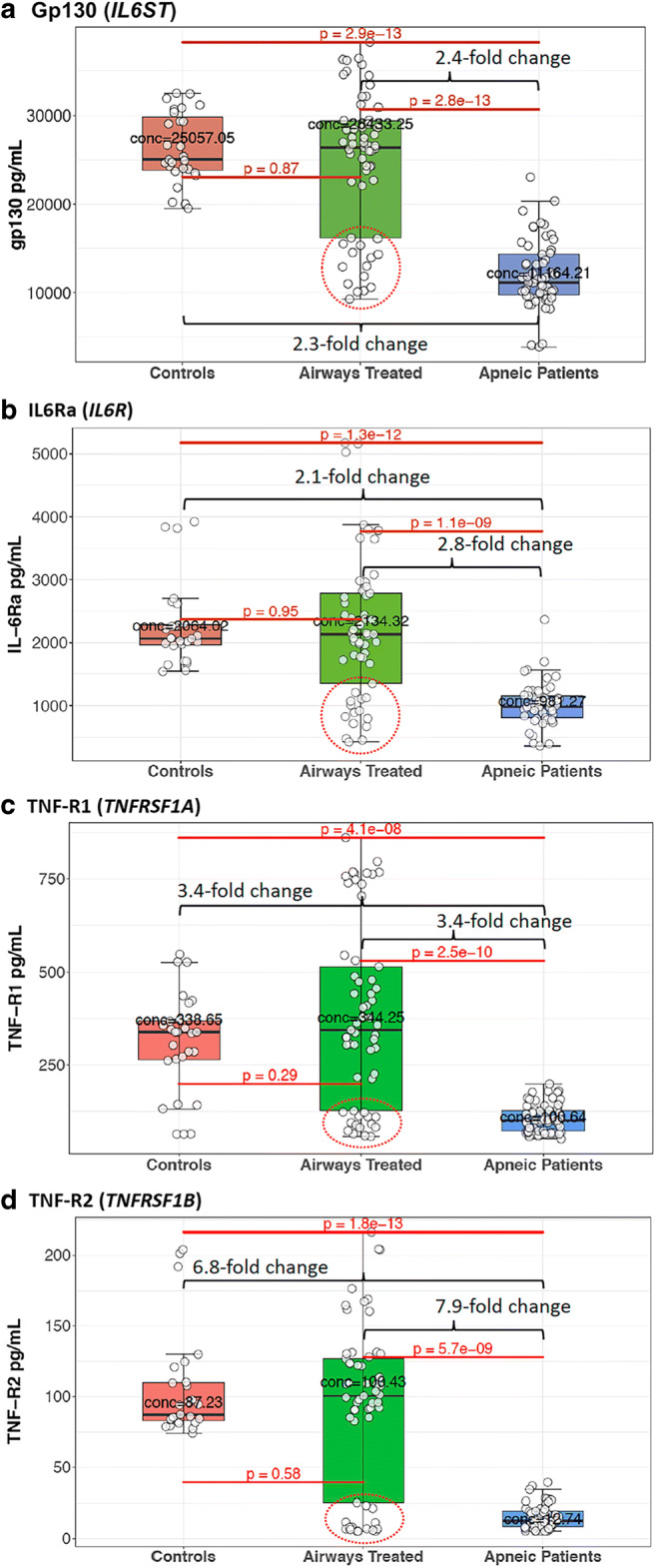
The levels of four cytokine receptors involved in neurodegenerative disease risk are low in OSA patients, but their levels in airway-treated OSA patients are indistinguishable from those in control subjects. The serum picogram per milliliter (pg/mL) levels of the soluble isoforms of **a** gp130, **b** IL6R, **c** TNFR1, and **c** TNFR2 for the eight control individuals, nineteen airway-treated apneic patients, and nineteen apneic patients not receiving airways therapy are summarized in box blots. The boxed area encloses the second and third quartile and is bounded by median pg/mL value indicated by a black line. The lower and upper whiskered ranges indicate the first quartile-1.5*IQR (interquartile range) and the third quintile +1.5*IQR, respectively. Each of the three independent Bio-Plex estimates of a cytokine level for each patient is represented by separate data points. Potential outlying data among airways treated patients are encircled by a red dotted line

Soluble serum cytokine receptor levels in airway-treated OSA patients were compared with untreated OSA patients and with control subjects. The picogram per milliliter (pg/mL) protein expression levels of the soluble isoforms of four inflammatory cytokine receptors known to be involved in ND, but not yet linked to OSA, were significantly lower in the serum of OSA patients relative to the levels for both the control individuals and the OSA patients receiving airways therapy. These data on the levels of soluble Gp130 (*IL6ST*), IL6R (IL6Ra, *IL6R*), TNF-R1 (*TNFRSF1A*), and TNF-R2 (*TNFRSF1B*) are shown in Fig. 1 and summarized in Table 2.

**Table 2:** Cytokine receptor levels among apneic patients and controls

Cytokine	Gene name	Control median pg/mL cytokine	Airways treated median pg/mL cytokine	OSA patients median pg/mL cytokine	Fold change from apneic to airways treated	Control vs OSA *p* value	Control vs airways treated *p* value	Airways treated vs OSA *p* value
gp130	*IL6ST*	25,057	26,433	11,164	2.37	2.92 × 10^−13^	0.867	2.77 × 10^−13^
IL-6Ra	*IL6R*	2064	2134	981	2.18	1.27 × 10^−12^	0.954	1.11 × 10^−9^
TNF-R1	*TNFRSF1A*	338	344	100	3.42	4.06 × 10^−8^	0.292	2.54 × 10^−10^
TNF-R2	*TNFRSF1B*	87	100	13	7.88	1.76 × 10^−13^	0.579	5.65 × 10^−9^

The median pg/mL cytokine levels of soluble gp130, IL6R, TNFR1, and TNFR2 are presented for airways-treated apneic patients, untreated apneic patients, and control individuals. The fold difference between the median level for airways-treated patients and untreated apneic patients are presented along with *p* values for all three pairwise comparisons of cytokine levels

The pg/mL concentrations of soluble gp130 and IL6R are shown as Box plot comparisons among the OSA patient population, OSA patients receiving airways therapy, and control individuals in Fig. 1a and b, respectively. It was observed that the median serum level of gp130 in OSA patients was 2.3-fold lower than that of the control subjects ((*p* = 2.9 × 10^−13^). OSA patients receiving airways treatment had a median level of gp130 that was much higher than that in untreated patients (*p* = 2.8 × 10^−13^), a median level indistinguishable from that observed in the younger control group (*p* = 0.87, Fig. 1a, Table 2). Similarly, the median serum level of soluble IL6R in OSA patients was 2.1-fold lower than in the serum of control subjects (*p* = 1.3 × 10^−12^). OSA patients receiving airways therapy had a median level of soluble IL6R that was 2.8-fold higher than that observed in untreated patients (*p* = 1.1 × 10^−9^), a level that was indistinguishable from that of the control group (*p* = 0.95, Fig. 1b).

The median pg/mL serum levels of soluble TNFR1 and TNFR2 were 3.4-fold and 6.8-fold lower in OSA patients relative to control subjects, *p* = 4.1 × 10^−8^ and 1.8 × 10^−13^, respectively (Fig. 1c and c, Table 2). The median serum level of TNFR1 was 3.4-fold higher in airway-treated patients than untreated apneic patients (*p* = 2.5 × 10^−10^, Fig. 1c) and was statistically indistinguishable from the median level in controls (*p* = 0.29). The median level of TNFR2 was 7.9-fold higher in airway-treated patients than untreated apneic patients (*p* = 5.7 × 10^−9^, Fig. 1c) and was also indistinguishable from the median level in controls (*p* = 0.58).

**Fig. 2:**
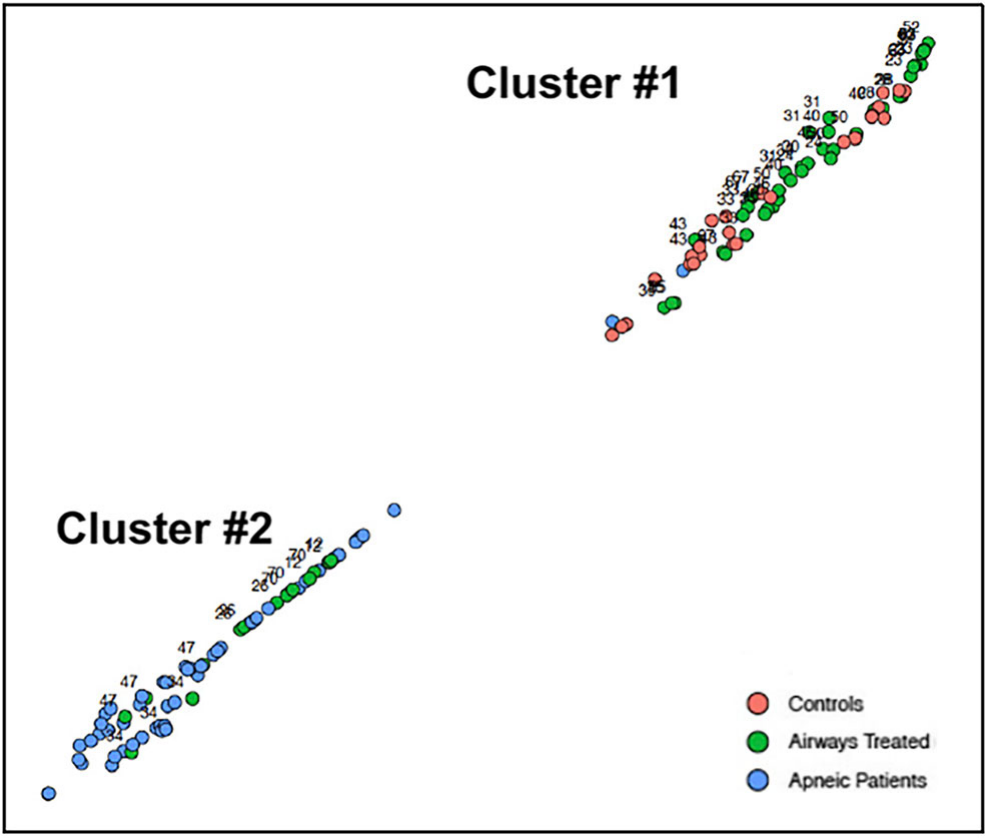
t-SNE clustered the cytokine receptor data for all OSA and airway-treated OSA patients and control individuals into two groups. Cluster 1 represents the dimensional distribution and grouping of data for four cytokines for all the control individuals, fifteen of the nineteen airway-treated OSA patients, and one untreated OSA patient. Cluster 2 represents the dimensional distribution of cytokine data among all but one of the untreated OSA patients and five of the airway-treated OSA patients. Each patient is represented by three separate data points. The individual patient numbers for the airway-treated OSA patient data are indicated in each cluster

In short, OSA patients had aberrantly and significantly low pg/mL levels of all four soluble cytokine receptors linked to ND relative to younger healthy control individuals. By contrast, OSA patients receiving airways therapy had cytokine receptor levels that were impossible to differentiate from those observed for control subjects. However, some of the data for five airway-treated patients (patient numbers 12, 26, 34, 47, and 70) were widely distributed and were more similar to levels in untreated OSA patients (see areas encircled by red dotted lines, Fig. 1). To better visualize the potentially similar response among most airway-treated individual patients independent of the direction or magnitude of change in the level of all four cytokines, the *t*-distributed stochastic neighbor embedding (t-SNE) method was applied. This machine-learning 2D visualization strategy has the capacity of capturing the local structure of these high-dimensional data and revealing the presence of data clusters as a global structure [110]. Figure 2 shows that all the patient and control subject cytokine receptor data group in two clusters. Cluster 1 represents the soluble cytokine receptor data for all nine control individuals (red data points in Fig. 2) and fourteen of the nineteen airway-treated patients (green data points in cluster 1). Cluster 2 represents the cytokine receptor data for all nineteen of the untreated OSA patients (blue data points in cluster 2) and five of the patients classified as airway treated (i.e., green data points within cluster 2). The cytokine receptor levels for patient numbers 12, 26, 34, 47, and 70 (numbered data points in Fig. 2) clearly distinguished themselves, because the combined levels of all four cytokines remain more similar to the levels in untreated OSA patients (again the encircled data points in Fig. 1).

The OSA patients, airway-treated patients, control subjects, and the outlying airway-treated OSA patient group did not appear to distinguish themselves based on a cursory analysis of biometric data such as age, BMI, gender, or particular chronic medications. A further analysis was made of the biostatistical, laboratory, and sleep data for potential causes for the variation in cytokine levels among all patients, controls, and particularly these five outlying airway-treated patients. Biometric, laboratory, and sleep data were collected at the time of serum collection for controls and for the untreated OSA patients and these data were collected months before serum collection at the time of their diagnosis of OSA for airway-treated patients. A linear regression analysis was performed, plotting the pg/mL level of each cytokine against age, BMI, heart rate, Apnea Hypopnea Index (AHI), and oxygen as SaO_2_ low %, ESS, and the laboratory data from duplicate serum samples including glucose levels, total cholesterol, HDL, LDL, and CRP. None of the biometric, laboratory, or sleep variables accounted for more than 40% of the variance in any of the cytokine levels among any of the any of the OSA patients, airway-treated OSA patients, control subjects or outliers, or all 49 subjects taken together. It was surprising that none of the sleep-related measures obtained correlated with cytokine levels in OSA. Although intermittent hypoxia is the hallmark of OSA, the findings in this study suggest that other mechanisms may be important in altering levels of GP130, IL6Ra, TNF-R1, and TNF-R2. One explanation for the nonparametric data for the small number outlying patients would be that their lack of complete adherence to airways treatment was misreported as adherence. The airways treated OSA patient using the dental airways device was not among the outliers, but had cytokine receptor levels similar to healthy controls.

## Discussion

We are working under a model in which chronic oxidative stress signaling in OSA patients alters the levels of soluble inflammatory cytokines in serum to initiate an inflammatory cascade that passes the blood brain barrier and increases the risk of ND. Making the presumption that cytokine levels respond to airways therapy, then aberrant levels in OSA patients may be directly or indirectly linked to oxidative stress signaling. The soluble, non-membrane-bound, isoforms of the membrane receptors gp130, IL6R, TNFR1, and TNFR2 all have been shown to regulate inflammatory signaling by their membrane-bound isoforms and by their cognate cytokines [78, 100]. Cytokines can penetrate the blood brain barrier particularly under circumstances of neuroinflammation [75]. If the significant reductions observed in the levels of all four soluble cytokine receptors in the serum of OSA patients pass the blood brain barrier, this should produce the mis-regulation of TNF and IL6 signaling in the brain. While this discussion is focused on the activities of the four cytokine receptors in ND, these receptors also have reported roles in autoimmune and cardiovascular diseases.

### IL6R and gp130

Altered levels of the membrane and/or soluble isoforms of gp130 and IL6R have been reported in the serum, cerebral fluid, and/or brain of patients with Alzheimer disease [112–114], amyotrophic lateral sclerosis [70], multiple sclerosis [71], and schizophrenia and bipolar disorder [72, 115]. A few single nucleotide polymorphisms affecting IL6R expression levels or protein activity also correlate with ND [70, 116–118]. Hence, it is reasonable to consider that the altered levels of soluble IL6R and gp130 observed in OSA patients are risk factors of ND and may even contribute causally to ND risk. Pro-and anti-inflammatory signaling by IL6 involving IL6R and gp130 is divided into two pathways. In the classical signaling pathway, IL6 binds to a classical receptor complex composed of the membrane isoforms of IL6R and gp130. Their inflammatory signaling is thought to contribute primarily to beneficial anti-inflammatory activities and tissue regeneration. In the trans-signaling pathway, both soluble and membrane isoforms of both cytokines are involved, and hence, their soluble isoforms have a greater potential to regulate trans-signaling. The trans-signaling pathway is thought to produce most of the harmful pro-inflammatory signaling leading to cell death directed by IL6 [78]. While the two signaling pathways were elucidated in non-neuronal systems and cell types [78], these are the best models for us to consider, while examining the roles of soluble IL6R and gp130 in neurodegeneration and ND.

Significant reductions in the levels of both soluble pg130 and soluble IL6R were observed in OSA patients relative to controls and relative to most airway-treated patients. Most airway-treated OSA patients in this study had serum levels of both cytokines that were essentially the same as controls. Because soluble gp130 is directly involved in attenuating harmful inflammatory trans-signaling by binding both the soluble and membrane isoforms of IL6R, the dramatic reductions observed in gp130 in OSA patients are likely to be harmful, increasing neuroinflammation, neurodegeneration, and ND risk [78]. However, it is harder to predict the consequences of reduced levels of soluble IL6R on ND, because soluble IL6R bound to IL6 binds with the membrane isoform of gp130 to enhance trans-signaling and IL6R binds to soluble gp130 with the potential to attenuate trans-signaling [78]. Previous studies had shown that the levels of the membrane isoforms of gp130 and IL6R were elevated by acute hypoxia in rodent models [119, 120], but the level of soluble IL6R was not altered in teenage OSA patients [58]. Finally, the data presented herein may be the first showing significant reductions in the levels of soluble gp130 and IL6R in the serum of older OSA patients and the normal levels of these cytokine receptors in patients receiving airways therapy.

There are immunotherapeutic approaches emerging to counter the reduction in gp130 and/or IL6R levels for OSA patients with symptoms of ND as a supplement to CPAP. Prenissl et al. [121] have shown that the monoclonal antibody tocilizumab, which specifically binds to the membrane receptor isoform of IL6R, increases the production of soluble IL6R twofold in peripheral tissues. Clinical studies have shown that tocilizumab significantly reduces symptoms of inflammatory disease, and it has been approved for the treatment of rheumatoid arthritis [122]. There is an ongoing clinical trial to treat depressed patients with tocilizumab [123]. However, the treatment of schizophrenic patients with tocilizumab did not improve patient behavior [124]. Assuming that reductions in the soluble isoforms of these receptors increase inflammatory signaling, then reducing IL6 activity is another alternative. Schuett et al. [125] showed treatment with modest concentrations of a fusion between the soluble isoform of gp130 and the IgG1 heavy chain constant region (sgp130Fc) dramatically reduced IL6 inflammatory signaling, presumably by substituting for soluble gp130 in trans-signaling. It has been proposed that sgp130Fc may be an effective therapeutic to treat depression [80, 126]. Unfortunately, inhibitors of IL6 itself, such as the IL6-specific monoclonal siltuximab, have not been particularly effective at inhibiting the symptoms of inflammatory disease [127], presumably because IL6’s activities are so pleiotropic.

### TNFR1 and TNFR2

The membrane and processed soluble isoforms of TNFR1 and TNFR2 both bind TNF [128, 129]. Altered expressions of one or both isoforms of these proteins are associated with Alzheimer’s disease [89–91], amyotrophic lateral sclerosis [32], depression [29, 86–88], Parkinson’s disease [85], and schizophrenia [93–96]. Both isoforms of TNFR1 and TNFR2 are involved in inflammatory signaling, but they generally signal via different transduction pathways and with nearly opposing outcomes [129]. TNFR1 predominantly promotes inflammation and neuronal cell death [83], while TNFR2 plays more neuroprotective roles in promoting cell survival and tissue regeneration [89]. The soluble isoforms of both are found in serum, and increasing their levels can antagonize or promote receptor signaling [98–100]. Hence, altering the ratio or levels of their co-expression can shift the balance between cellular survival, regeneration, and apoptosis. The expression of both receptors increases when cultured cells are treated with acute intermittent hypoxia [130], but in vitro studies examining the impact of chronic hypoxia have not been reported.

Previous studies yielded marginal or conflicting results concerning the levels of the soluble isoforms of TNF receptors in OSA patients, with TNFR1 levels reported as 2.1-fold higher than the levels control subjects [131] or only 1.1-fold higher [132] or indistinguishable from controls [58, 133]. An earlier report showed that airways therapy of OSA patients decreased plasma levels of soluble TNFR1 marginally 1.2-fold relative to untreated OSA patients [134]. Finally, one study showed that OSA patients with AHI scores higher than 10 had 1.5-fold higher levels of soluble TNFR1 and TNFR2, relative to patients with an AHI lower than 10 [135]. Hence, both the direction and small magnitude of change in these studies of OSA patients are inconsistent with the data collected herein. Although, deficiencies in soluble TNFR2 have been associated with inflammatory autoimmune diseases [136].

By contrast to most previous studies, herein, statistically significant several-fold lower levels of soluble TNFR1 and TNFR2 were observed in untreated OSA patients relative to control subjects. Cytokine receptor levels were dramatically higher in airway-treated OSA patients and were essentially the same as controls. These results strongly suggest that the serum levels of the soluble isoforms of both cytokines are positively regulated by blood oxygen levels. Consistent with these data, chronically increased TNF expression has been associated with reduced levels of TNFR1 and TNFR2 in the brain [97, 137]. Perhaps the chronic intermittent hypoxia experienced by OSA patients creates a chronic increase in TNF, with the potential to lower soluble TNFR1 and TNFR2 levels.

It is worth considering why such large, highly statistically significant changes were observed herein in the levels of TNFR1 and TNFR2 as compared with the small 1.2- to 1.5-fold changes observed in other studies of OSA patients relative to controls or to the airway-treated OSA patients. The fluorescent bead immunocapture microfluidic technology applied to this patient population gives highly dynamic results based on hundreds of fluorescent beads for each measurement of pg/mL cytokine levels, instead of using less dynamic colorimetric assay in a few wells of a microtiter plate provided by ELISA kits [131] such as reported in most earlier studies. Fluorescent bead immunocapture microfluidics is more sensitive or at least as sensitive as and statistically reproducible as ELISA microtiter plate measurements of cytokines used in these earlier studies [138, 139] and fluorescence bead assays have a higher dynamic range. The standard errors among the three independent replicate assays performed for each cytokine were very small, even for the lowest concentrations observed in untreated OSA patients (see Materials and methods). It appears that fluorescent bead capture technology provided an advantage, when assaying the dynamic changes observed in cytokine levels among apneic patients and airway-treated apneic patients and control subjects.

One reasonable interpretation of these results would be that the aberrantly low levels of soluble TNFR1 and TNFR2 in OSA patients might no longer provide appropriate attenuation of TNF inflammatory signaling, thus increasing the risk of ND. A few therapeutic treatments have been shown to specifically block membrane TNFR1 signaling or to increase TNFR2 activity [97]. ATROSAB is a humanized mouse monoclonal to TNFR1 that covers the TNF binding epitope and neutralizes TNFR1’s membrane signaling activity [140]. ATROSAB prevents neuronal cell death in a mouse model of cognitive impairment and ND [141]. Whereas in the same model, simultaneously blocking both TNFR1 and TNFR2 proved ineffective. ATROSAB also proved to be an effective treatment for multiple sclerosis in a mouse disease model [142]. Perhaps therapeutic treatment of OSA patients with ATROSAB could aid OSA patients with symptoms of ND, as a supplement to CPAP. Finally, the cholesterol-lowering small molecule drug, lovastatin, selectively increases TNFR2 expression [143] and prevents cognitive deficits in mice [144].

## Conclusions

The serum levels of the soluble isoforms of four membrane receptors, gp130, IL6R, TNFR1, and TNFR2, with roles in attenuating inflammatory signaling and neuronal cell death, and hence, risk factors for ND, were examined. The pg/mL protein levels of all four were expressed at aberrantly low levels in OSA patients. The majority of airway-treated OSA patients had levels for all four soluble cytokine receptors equivalent to those observed in healthy control individuals. In short, airways treatment was strongly correlated with normal cytokine receptor levels in most of the airway-treated OSA patients. This correlation with airways treatment suggests that chronic intermittent hypoxia may be among the factors contributing to the aberrantly low expression of TNF and IL6 receptors in untreated OSA patients. Several other OSA-associated abnormalities such as sleep fragmentation and daytime sleepiness may also play a role in altering the expression of cytokines and cytokine receptors. Furthermore, any conclusions drawn from this study are partially compromised by the surprising lack of a correlation between cytokine receptor levels and CRP levels, SaO2 low percent, and perhaps the relatively young age of our control subjects. Increasing the serum levels of these cytokines in OSA patients may be a beneficial supplement to airways therapy.

## Electronic supplementary material

ESM 1  (XLSX 179 kb)

## Data Availability

See Supplementary Data File 1.

## Abbreviations The gene names for cytokines are shown in italic

ND: Neurodegenerative disease
OSA: Obstructive sleep apnea syndrome
CPAP: Continuous positive airways pressure
t-SNE: *t*-distributed stochastic neighbor embedding
gp130 (*IL6ST*): Interleukin 6 signal transducer, membrane glycoprotein 130
IL6R (*IL6R*): IL-6R, IL6Ra, interleukin 6 receptor
TNFR1 (*TNFRSF1A*), TNF-R1: Tumor necrosis factor receptor superfamily member 1A
TNFR2 (*TNFRSF1B*) TNF-R2: Tumor necrosis factor receptor superfamily member 1B
TNF (*TNF*): TNF-α, tumor necrosis factor ligand superfamily member 2
IL6 (*IL6*): IL-6, interleukin 6, B cell stimulatory factor 2
